# Combination of FACS and Homologous Recombination for the Generation of Stable and High-Expression Engineered Cell Lines

**DOI:** 10.1371/journal.pone.0091712

**Published:** 2014-03-19

**Authors:** Lei Shi, Xuesi Chen, Wenying Tang, Zhenyi Li, Jin Liu, Feng Gao, Jianli Sang

**Affiliations:** 1 Key Laboratory of Cell Proliferation and Regulation Biology, Ministry of Education, College of Life Sciences, Beijing Normal University, Beijing, China; 2 R&D Department, AutekBio, Inc., Beijing, China; Chang Gung University, Taiwan

## Abstract

Traditionally, cell line generation requires several months and involves screening of over several hundred cell clones for high productivity before dozens are selected as candidate cell lines. Here, we have designed a new strategy for the generation of stable and high-expression cell lines by combining homologous recombination (HR) and fluorescence-activated cell sorting (FACS). High expression was indicated by the expression of secreted green fluorescent protein (SEGFP). Parental cell lines with the highest expression of SEGFP were then selected by FACS and identified by stability analysis. Consequently, HR vectors were constructed using the cassette for SEGFP as the HR region. After transfecting the HR vector, the cells with negative SEGFP expression were enriched by FACS. The complete exchange between SEGFP and target gene (TNFR-Fc) cassettes was demonstrated by DNA analysis. Compared with the traditional method, by integrating the cassette containing the gene of interest into the pre-selected site, the highest producing cells secreted a more than 8-fold higher titer of target protein. Hence, this new strategy can be applied to isolated stable cell lines with desirable expression of any gene of interest. The stable cell lines can rapidly produce proteins for researching protein structure and function and are even applicable in drug discovery.

## Introduction

In recent years, the market for global biopharmaceuticals has widely expanded, and it is expected to exceed sales of US $166 billion by 2017 [1]. Major pharmaceutical products are recombinant proteins that are produced in cultivated mammalian cell lines, among which the Chinese hamster ovary (CHO) cell line is used to produce almost 70% of all recombinant protein therapeutics [2], [3]. In the process of recombinant protein production, one of the critical steps is rapid selection of stable and high-expression cell lines for the gene of interest (GOI), which is a time-consuming and labor-intensive step [4]. To generate cell lines for the production of target proteins, the traditional strategy involves transfection of the target gene for random integration into genomic DNA by homologous recombination (HR). The titer of the target protein is then analyzed among a large number of cell clones to select high-expression cell clones. Using this method, more than 80% of cell clones express the GOI at a very low level. Even in high-expression cell clones, GOI expression needs to be increased by several rounds of amplification. Lastly, single cell clones can be isolated by subcloning [5], [6]. Furthermore, the selected cell clones have some limitations, such as instability and/or slow cell growth [7]. The most important step of this procedure is integration of the GOI into a stable and high-expression site in the genomic DNA, which enables high and continuous expression of the GOI. Therefore, in modern biopharmaceutical technology, different strategies have been developed to increase the screening throughput of cell clones and/or raise GOI expression directly.

More than 100 million cells are used to establish one cell line for recombinant protein production [6]. To obtain more cell clones, many more cells need to be analyzed and rapidly selected by high-throughput screening. Fluorescence-activated cell sorting (FACS) is a widely used method for rapid analysis of a large number of cells [8]. There are several strategies that can be applied to this technology: 1) green fluorescent protein (GFP) as a reporter gene for selection of GOI high-expression cells [9]; 2) immunostaining using an antibody or Fc-fusion protein and sorting the highly fluorescent cells that indicate high-expression cells [10]; 3) selection of a new host cell line from a large number of cells to generate the GOI high-expression cell line [11], [12]. On the other hand, cell clones can be analyzed by flow cytometry at the early stage to determine their stability [13].

Very different strategies have been developed to increase GOI expression, including insertion of an increased expression element or using a new promoter to increase transcription of the GOI [14], [15]. These strategies include using STAR/MARs/UCOE elements to reduce gene silencing induced by epigenetic effects [16]–[18], selection of cell lines containing a hotspot region for high expression, as indicated by a reporter gene, and integration of the GOI into these regions using Cre-LoxP and/or Flp-In systems [19], [20]. All of these strategies would save time and reduce costs to obtain high-expression cell lines.

In this study, we report a new strategy for establishment of a GOI high-expression cell line. By combining HR and FACS, our strategy was designed to enrich and collect the gene-replaced cells that exchanged a secreted GFP (SEGFP) cassette with the GOI cassette at a hotspot in the genome. Compared with the traditional method, our results revealed that the titer of GOI-encoded protein increased approximately 8-fold by insertion of the GOI into the pre-selected site. The GOI-engineered cell lines inherited the stable cell growth and protein expression of the parental cell line. Therefore, to construct stable and high-expression cell lines, only a small number of cell clones needs to be generated and analyzed using our strategy, which saves time and significantly reduces costs. Considering these advantages, combining HR and FACS provides a new and rapid strategy for the generation of stable and high-expression cell lines.

## Materials and Methods

### Plasmids

SEGFP contains a signal peptide from the human IgG kappa chain in the N-terminal. The expression cassette was composed of a CMV promoter, SEGFP coding region, and BGH polyA sequence.

To construct a TNFR-Fc expression control plasmid, a DNA fragment of the human EF1α promoter and TNFR-Fc gene was amplified by PCR from the proprietary TNFR-Fc expression vector. The amplified DNA fragment was digested with PvuI/EcoRI, and then inserted into a pBudCE4.1 plasmid (Invitrogen, USA) to replace the CMV promoter.

All HR plasmids were constructed from the control vector. For HR plasmid A, the full-length CMV sequence and 400 bp SEGFP N-terminal region were amplified, digested by PvuI, and then inserted upstream of the human EF1α promoter. The 300 bp SEGFP C-terminal sequence and BGH polyA signal sequence were cloned and inserted downstream of the zeocin-resistance gene after digestion by NheI.

HR plasmid B was obtained by ligating the NheI-digested *SEGFP c-polyA-Neo p* fragment into digested plasmid A. The *SEGFP c-polyA-Neo p* fragment contained the SEGFP C-terminal sequence region (300 bp) to the partial Neo gene.

HR plasmid C was also derived from plasmid A, in which the upstream HR element was replaced by a partial CMV promoter (CMV p; 200 bp).

### Cell Culture

CHO-S cells were purchased from Invitrogen and cultivated in CD-CHO medium (Gibco/Invitrogen, USA) containing 8 mM L-glutamine and HT supplement (100 mM hypoxanthine and 16 mM thymidine). Host cells and cell clones were cultured in 100 ml shaking flasks containing 20 ml medium at 37°C in a 5% CO_2_ incubator. Cell concentration and viability were measured by trypan blue exclusion using a hemocytometer. Routine passaging was performed at a frequency of three times per week. SEGFP cell clones were cultured under the same condition with growth medium containing 0.6 mg/ml G418. Recombinant cell pools and clones were cultured in medium containing 0.1 mg/ml zeocin.

Population doubling time (PDT) was calculated from the culture time versus a semi-log plot of the viable cell concentration in the exponential growth phase by the following formula:
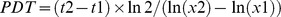
where x_1_ and x_2_ are the viable cell concentrations at time points t_1_ and t_2_.

Integral cell area (ICA) was calculated by the following equation:
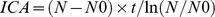
where N and N_0_ represent the final and initial viable cell numbers, respectively, while t represents the days in culture.

### Genes Transfer

#### Electroporation

CHO-S cells were seeded at 24 h before transfection. A total of 5×10^6^ cells and 10 μg DNA were used for transfection at 350 V for 30 ms in a Gene-Pulse (Bio-Rad, USA). The transfected cells were resuspended in CD-CHO medium and cultured in a shaking flask with 10 ml of culture medium. For selection of cells as a control, at 1 day post-transfection, the electroporated cells were plated in 24-well plates with culture medium containing 0.1 mg/ml zeocin.

#### Transfection of HR plasmids

As described above, at 1 day prior to transfection, SEGFP-transfected cell clones were seeded in a shaking flask. A total of 5×10^6^ logarithmically growing cells were electroporated with HR plasmids (10 μg). The transfected cells were recovered in a shaking flask containing growth medium. At 72 h after transfection, transfected cells were collected for SEGFP-negative cell sorting.

### Measurement of SEGFP and TNFR-Fc

#### Fluorescence Intensity (FI) measurement

Culture medium of each SEGFP cell clone was collected and centrifuged at 13,000 g for 10 min to remove cell debris. The medium supernatant was then transferred to a fresh 1.5-ml tube. A total of 200 μl medium supernatant was aliquoted into 96-well plates. Fresh medium was used as a blank control. The plates were then scanned on a Cytofluorescent Synergy™ 2 Fluorescence Measurement System (Biotek, USA) with filter sets covering the GFP excitation and emission wavelengths (excitation: 485±20 nm; emission: 530±25 nm).

The quantitative FI (QFI) was calculated with the FI by the following equation:
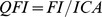
where FI is the total FI in the culture supernatant as determined by spectrofluorometric measurement.

#### Enzyme-linked immunosorbent assay

To determine TNFR-Fc titers, conditioned medium was analyzed by the sandwich enzyme-linked immunosorbent assay method. Essentially, 96-well plates (Constar/Corning, USA) were pre-coated with a goat anti-human IgG primary antibody (CW bio, China) at 37°C overnight. Following a series of wash steps, the wells were blocked with bovine serum albumin in PBS and Tween 20. Bound recombinant antibodies were detected using TMB substrate (Invitrogen, USA) following incubation of each well with a mouse anti-human IgG HRP-conjugated secondary antibody (CW bio, China). Following 30 mins of incubation at room temperature, the TMB substrate reaction was terminated with 2 M H_2_SO_4_ prior to reading at 450 nm using a microplate reader (Thermo, USA). Data were collected and analyzed using GraphPad Prism 5. Quantification was based on a dilution series to obtain a standard curve of known TNFR-Fc concentrations.

#### Western blotting

SEGFP and TNFR-Fc proteins in culture supernatants were analyzed by western blot. An aliquot of growth medium was separated by sodium dodecyl sulfate-polyacrylamide gel electrophoresis. The proteins were then transferred onto a polyvinyl difluoride membrane, and the presence of SEGFP or TNFR-Fc was demonstrated using anti-GFP (CW bio, China) and anti-human IgG antibodies, respectively, following by DAB staining (Tiangen, China).

### Flow Cytometry

Cells prepared as described above were resuspended in cold PBS at approximately 1×10^6^ viable cells/ml, followed by analysis using a FACSCalibur flow cytometer (BD Biosciences, USA). Auto fluorescence of untransfected cells was detected as a control. Data acquisition was carried out by analyzing 10,000 events/sample using Cell Quest Software (BD Biosciences, USA).

### FACS

At 3–4 days after transfection, exponentially growing cells were harvested and resuspended in culture medium with 1% penicillin/streptomycin at approximately 5×10^6^ viable cells/ml. The collected cells were then sorted on a FACStar PLUS. Live cells were gated based on forward/side scatter and events in the top 3–4% of GFP fluorescence were sorted. Seven days later, a second round of cell sorting was performed to collect cells in the top 1–2% of GFP fluorescence. The sorted SEGFP high-expression cells were cultured in medium containing 0.6 mg/ml G418.

For HR cells, fluorescently gated cells in the lowest 5–6% (first round) and 2–3% (second round) were sorted. Both groups of sorted cells were cultured in growth medium with 0.1 mg/ml zeocin. SEGFP high-expression cells and HR cells from the second round of sorting were expanded and subcloned at a concentration of 1 cell/well in 96-wellplates.

### DNA Analysis and Copy Number Measurement

Genomic DNA was harvested from cells with a Genomes DNA extraction Kit (TaKaRa, Japan). PCR was performed with HS Primer STAR polymerase (TaKaRa, Japan) according to the manufacturer’s instructions using a MJ (Bio-Rad, USA) as follows: pre-denaturation at 94°C for 3 min, followed by 35 cycles of denaturation at 94°C for 30 s, annealing at 65°C for 1 min, and elongation at 72°C for 2 min. The primers used were as follows:

CMV-S: 5′ GTACATTTATATTGGCTCATGTCCAACATTACCGC 3′

SEGFP-A:5′ GGCGGACTGGGTGCTCAGGTAGTGGTT 3′

TFNR-A:5′ AAGATTTGGGCTCAACGCTACCTCCACC 3′

DNA was extracted from cells using an All Prep DNA Mini kit (Qiagen, Germany), and then applied to qPCR (SYBR® Premix Ex Taq™ kit; TaKaRa, Japan) to determine the copy number of the SEGFP gene using the following primers: forward: 5′ TGCTTCAGCCGCTACCC 3′ and reverse: 5′ TCACCTTGATGCCGTTCTT 3′. PCR was performed on an Opticon monitor II (Bio-Rad, USA) at 95°C for 30 s, followed by 45 cycles of 95°C for 5 s and 60°C for 30 s. The linearized SEGFP vector was used to generate a standard curve, and copy numbers were determined using the absolute quantitative method.

## Results

**Figure 1:**
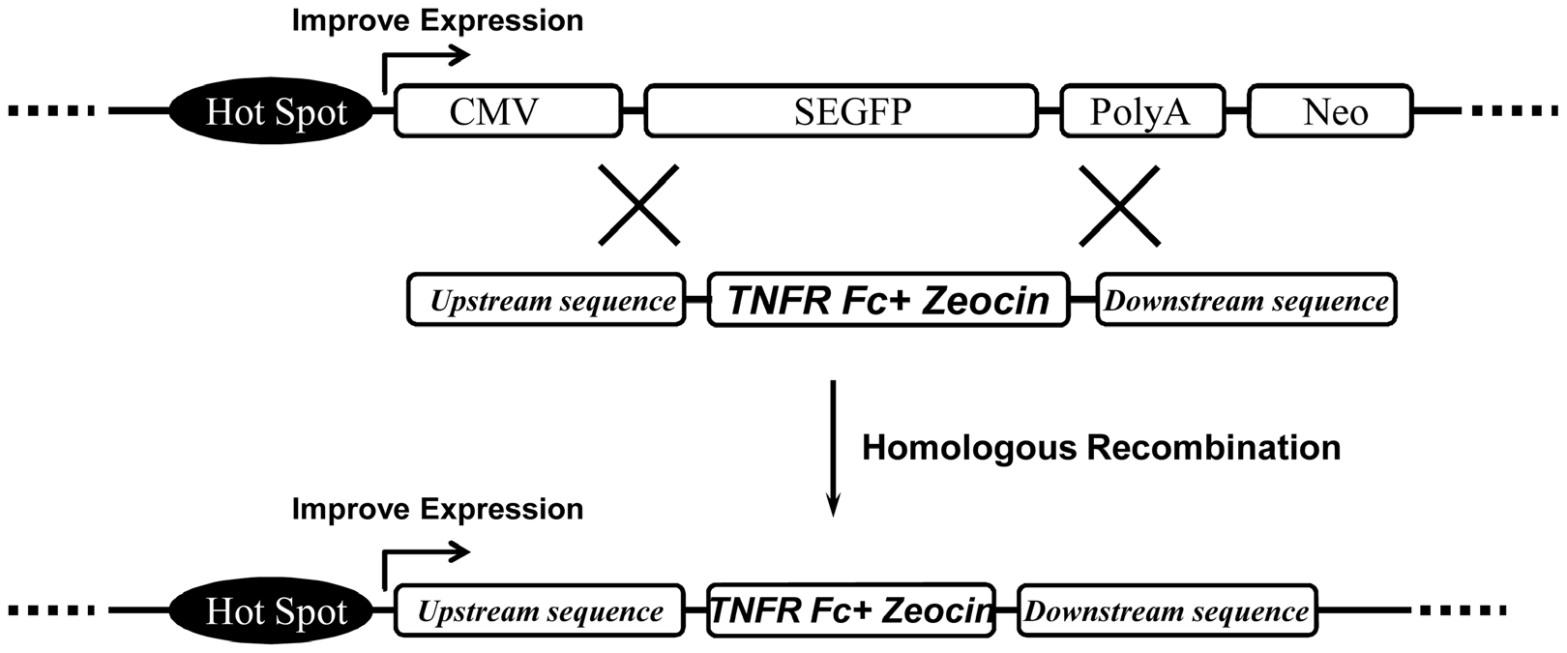
Schematic diagram of the HR strategy. A hotspot region was indicated by high expression of SEGFP. The SEGFP gene would be replaced with the GOI though HR. The GOI could then be highly expressed under the control of the hotspot region.

In the pharmaceutical industry, establishing a stable and high-expression cell line is very labor-intensive and highly inefficient. To improve the traditional method, we considered generation of a high-expression cell line based on HR. As shown in Fig. 1, to obtain the GOI high-expression cell line, high-expression parental cells were first selected using SEGFP as a marker. A high expression level of SEGFP indicated that its insertion site was a hotspot region for exogenous protein expression. In addition, the SEGFP expression cassette was used as the homologous sequence to construct the HR vector. In the HR vector, the CMV promoter-SEGFP N-terminal sequence and SEGFP C-terminal-polyA signal sequence were inserted into the upstream and downstream sequence of the GOI expression cassette, respectively. According to this design, the GOI would be inserted in the pre-selected site by replacing the SEGFP expression cassette with the HR region to easily establish the GOI high-expression cell line. This strategy included the following key points: selection of SEGFP high-expression cell lines, construction and functional analysis of HR vectors, collection of SEGFP-negative cells, and measurement of GOI expression in replaced cells (SEGFP-negative cells).

### SEGFP Parental Cell Line Generation

**Figure 2:**
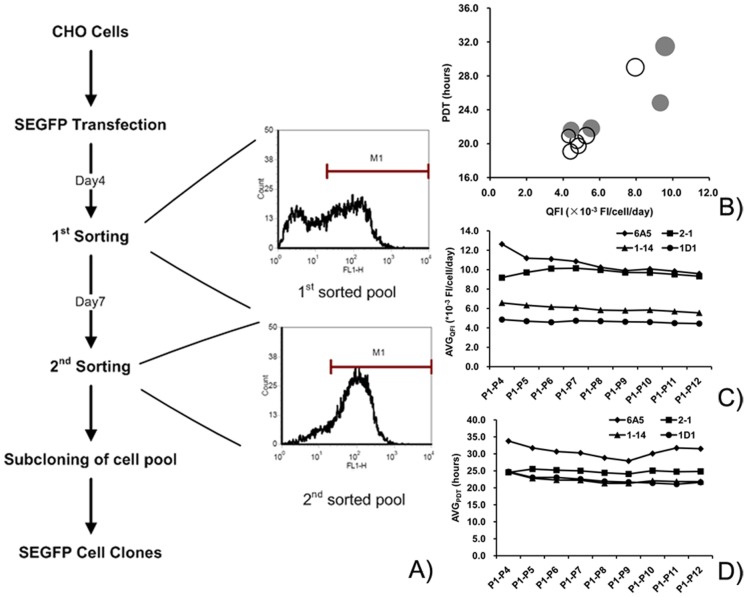
SEGFP parental cell line generation by FACS and identification of stability. (A) Flow chart of selection of SEGFP cell clones. CHO cells were transfected with the SEGFP expression vector, and then collected by two rounds of FACS. The profiles of flow cytometry showed enrichment of SEGFP high-expression cells. (B) Comparison of the top 10 SEGFP cell clones. According to the QFI and FI values, four clones (gray circles) were selected to analyze stability. (C, D) Stability analysis of SEGFP expression and growth of the four cell clones. The average QFI and PDT were calculated and compared to indicate the variation of SEGFP expression and cell growth, respectively.

The most important point of this strategy is selection of a parental cell line with high expression of the reporter gene. SEGFP was used as a marker to select the high-expression cells by two rounds of FACS. Cells with the highest expression of SEGFP (top 3–4%) were sorted on day 4 after transfection. After 7 days, the top 1–2% of cells with high expression of SEGFP were sorted again (Fig. 2A). After the sorting process, almost all sorted cells were SEGFP positive. The percentage of SEGFP high-expression cells was increased from 81.8% (first-sorted cells) to 99.1% (final sorted cells).

To select a SEGFP high-expression parental cell line, the cells from the second round of FACS were individually cultured in 96-well plates. After comparing the expression of SEGFP, the top 10 clones were chosen from among 285 clones to analyze the stability of cell growth and SEGFP expression. Fig. 2B shows the relationship of quantitative fluorescence intensity (QFI), Population doubling time (PDT) and total FI among these clones. The average PDT of the SEGFP clones ranged from 19 to 22 h, except for clones 6A5 (29.4 h), 20E2 (29.02 h) and 2–1 (24.83 h). Consistent with their PDTs, the average QFI was higher in the three clones than that in other clones. Average QFI values among these clones had a more than 2-fold divergence from 4.3 to 9.58×10^−3^ FI/c/d. However, the discrepancy was only 25% between the highest and lowest FI. Furthermore, all SEGFP clones were single-positive clones as determined by flow cytometry. The coefficients of variations (CV) in different clones ranged from 4.3 to 7.5 (data not shown).

Combined with QFI and total FI, four SEGFP clones (gray circles in Fig. 2B: 6A5, 2-1, 1–14, and 1D1) were selected to carry out the HR experiment. The characteristic parameters of the four SEGFP clones are summarized in Table 1, including PDT, QFI, percentage of positive cells (positive %), CV, and copy number. The four clones showed two different sets of characteristics: 6A5 and 2-1 cell clones grew slower with a higher QFI and lower uniformity, whereas 1–14 and 1D1 cell clones grew faster with a lower QFI and higher uniformity. Moreover, the stability of these clones in terms of cell growth and SEGFP expression was analyzed by the average PDT (PDT_AVG_) and average QFI (QFI_AVG_), respectively. The PDT_AVG_ of these clones changed smoothly, indicating that these clones had stable growth over multiple passages (Fig. 2C). The variation of QFI_AVG_ revealed that the SEGFP expression levels in 2-1, 1–14 and 1D1 cell clones were stable at the start of subculture. After adaption to the shaking flask, SEGFP was expressed stably in the 6A5 cell clone beginning from passage 8 (Fig. 2D).

**Table 1:** Comparison of the four SEGFP cell clones.

Clone#	PDT (hours)^a^	Qp(*10^−3^ FI/c/d)^b^	Positive%^c^	CV^d^	Copy Number
6A5	29.40±10.2	9.58±2.9	100.00%	6.71	1.2±0.2
2-1	25.16±4.5	9.32±1.7	98.20%	7.38	1.2±0.1
1–14	21.79±5.4	5.54±1.1	99.90%	4.82	1.7±0.2
1D1	21.60±4.6	4.44±0.8	99.80%	4.33	0.9±0.1

a,b Average PDT and QFI from passage 1 to 12.

c Positive cells were defined as cells with a 2-fold higher FI than the highest FI in the negative control.

d Coefficients of variations of positive cells in the cell population.

### Construction of HR Vectors

**Figure 3:**
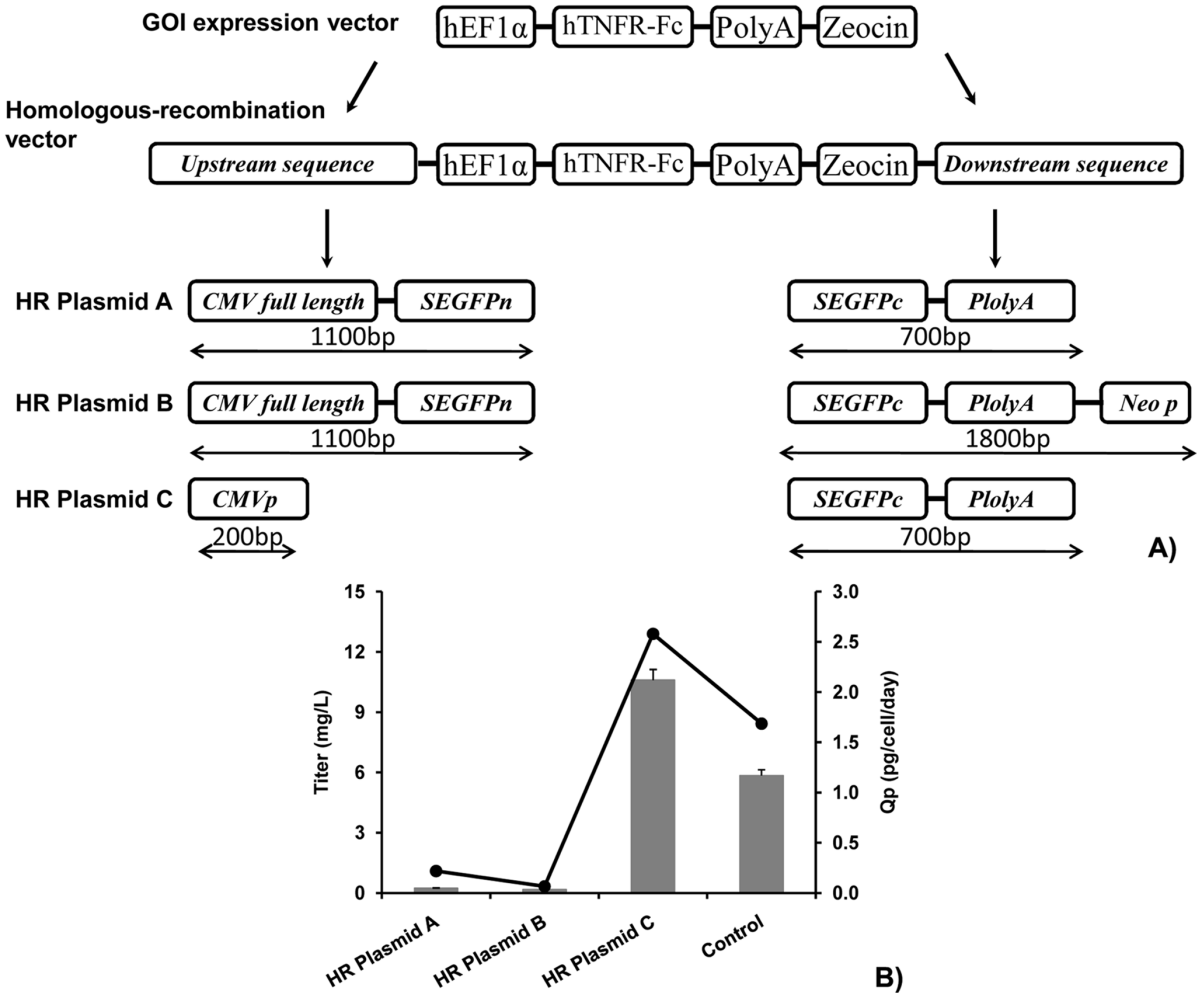
Construction and GOI expression of HR vectors. (A) Schematic diagram of HR vectors. Three HR vectors were constructed from the TNFR-Fc expression vector. Fragments of the SEGFP expression cassette with different lengths were used for HR in the upstream and downstream sequences of the TNFR-Fc expression cassette. (B) HR vectors were transiently transfected into CHO cells using the TNFR-Fc expression vector as the control. The titers and Qp of TNFR-Fc were measured and compared among these vectors.

In parental cells, the SEGFP cassette from the CMV promoter to the polyA signal sequence had to be inserted into the genomic DNA. Therefore, HR vectors derived from the TNFR-Fc expression vector were constructed by inserting the homologous sequence of the SEGFP gene into the upstream and downstream sequence of the GOI (Fig. 3A).

Three different vectors were constructed for HR. Plasmid A contained the full-length CMV promoter and a partial sequence of the SEGFP N-terminal (1100 bp) in the upstream and a partial sequence of the SEGFP C-terminal plus polyA region (700 bp) in the downstream. The downstream element of plasmid B was increased to 1700 bp by adding a partial Neo gene sequence behind the polyA region, and the upstream sequence had no changes from plasmid A. In plasmid C, the size of the upstream element was decreased to 200 bp, which only contained a partial CMV promoter, and the downstream element remained unchanged from plasmid A.

First, the plasmid construction should not inhibit GOI expression. To detect the expression of the GOI (TNFR-Fc) from the different HR vectors, we transfected these plasmids into CHO cells and used the TNFR-Fc expression vector as a control. After electroporation, the cell growth of transfected cells remained the same. However, compared with the TNFR-Fc titer produced by the control vector (6 mg/L), only plasmid C could express a higher TFNR-Fc titer (10 mg/L). The Qp of plasmids A and B was also much lower than that of the control (Fig. 3B). Thus, plasmid C was used for HR.

### HR for Site-specific Integration of the GOI

#### Selection of SEGFP-negative cells by FACS and comparison of sorted cell pools

**Figure 4:**
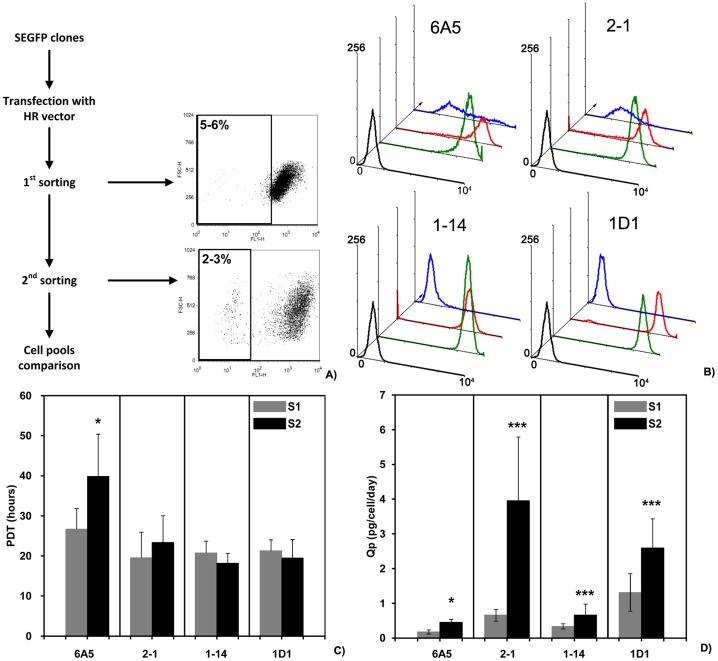
SEGFP-negative cells sorted by FACS and analysis of sorted cell pools. (A) An overview of sorting SEGFP-negative cells. After transfection with HR plasmid C, the four SEGFP cell clones were sorted by FACS twice. The FACS profiles showed the sorting cell population and the percentage was the sorting ratio in different sorting steps. (B) The sorted cell pools were analyzed and compared with the corresponding SEGFP cell clones. Using CHO cells (blank line) as a control, FACS profiles showed the fluorescence signals from SEGFP cell clones (green line) compared with those of the cell pools from the first (red line) and second (blue line) rounds of cell sorting. Although SEGFP-negative cells were only collected from 1D1 and 1–14 cell clones, the cell size and the protein content of all four sorted cell pools (-S2) did not change from that of each parental cell line (Supplemental Figure 1). (C, D) The cell growth of sorted cell pools remained consistent according to comparison of PDTs. However, TNFR-Fc expression was increased in every sorted cell pool following the sorting process.

According to the design, the SEGFP gene would be replaced by the GOI expression cassette after transfection of HR plasmids. Thus, we collected SEGFP-negative cells, even though not all SEGFP-negative cells represented replaced cells. The flow chart in Fig. 4A shows the two-round selection procedures to obtain SEGFP-negative cells by FACS. In the first round of sorting, cells in the lowest 5–6% of SEGFP expression were selected and designated as the “-S1” cell pool. After recovery in 24-well plates, the 2–3% SEGFP lowest-expression cells were then sorted and designated as the “-S2” cell pool.

The cell pools were expanded and analyzed. In all four cell clones, the SEGFP lower-expression cells were enriched after the two rounds of sorting (Fig. 4B). However, the cell pools selected from different parental cell lines had a different pattern of SEGFP expression. For example, the first cell pools (1D1-S1 and 1-14-S1) derived from 1D1 and 1–14 cells consisted of the lower 5–6% SEGFP-negative cells and almost all cells were SEGFP negative in the second cell pools (1D1-S2 and 1-14-S2). In contrast, SEGFP expression in 6A5-S2 and 2-1-S2cell pools was also decreased significantly, but SEGFP-negative cells were not enriched by FACS. The cell size and protein content were also compared between sorted cells and parental cells by flow cytometry (Fig. S1). No significant variations were observed among sorted cell pools and parental cell lines, although the four cell clones had different cell sizes and protein contents.

In addition to comparison of SEGFP expression, cell growth and TNFR-Fc expression were compared in sorted cell pools (Fig. 4C and 4D). The average PDTs of 2-1-S2, 1-14-S2 and 1D1-S2 cells were unchanged, compared with that of the first-sorted cell pools. All of the cell pools had PDTs of approximately 20 h, suggesting that the cells selected by FACS could grow very well. However, the 6A5-S2 cell pool had a significantly longer PDT (39.84 h) than that of the 6A5-S1 cell pool (26.67 h) (P = 0.026). Following FACS, the average Qp of TNFR-Fc was increased compared with that of the corresponding cell pools derived from different cell clones. The Qp of 1D1-S2 and 1-14-S2 cell pools was 2.60 and 0.66 pg/cell/day, respectively. Although 2-1-S2 cells were SEGFP negative, the Qp of TNFR-Fc in the cell pool increased to 3.96 pg/cell/day. These results demonstrated that SEGFP-negative or lower-expression cells can be collected and enriched by FACS, the SEGFP-negative cells expressed TNFR-Fc, and the Qp of TFNR-Fc increased along with the increase of SEGFP-negative cells.

#### Site-integration analysis of HR cells

**Figure 5:**
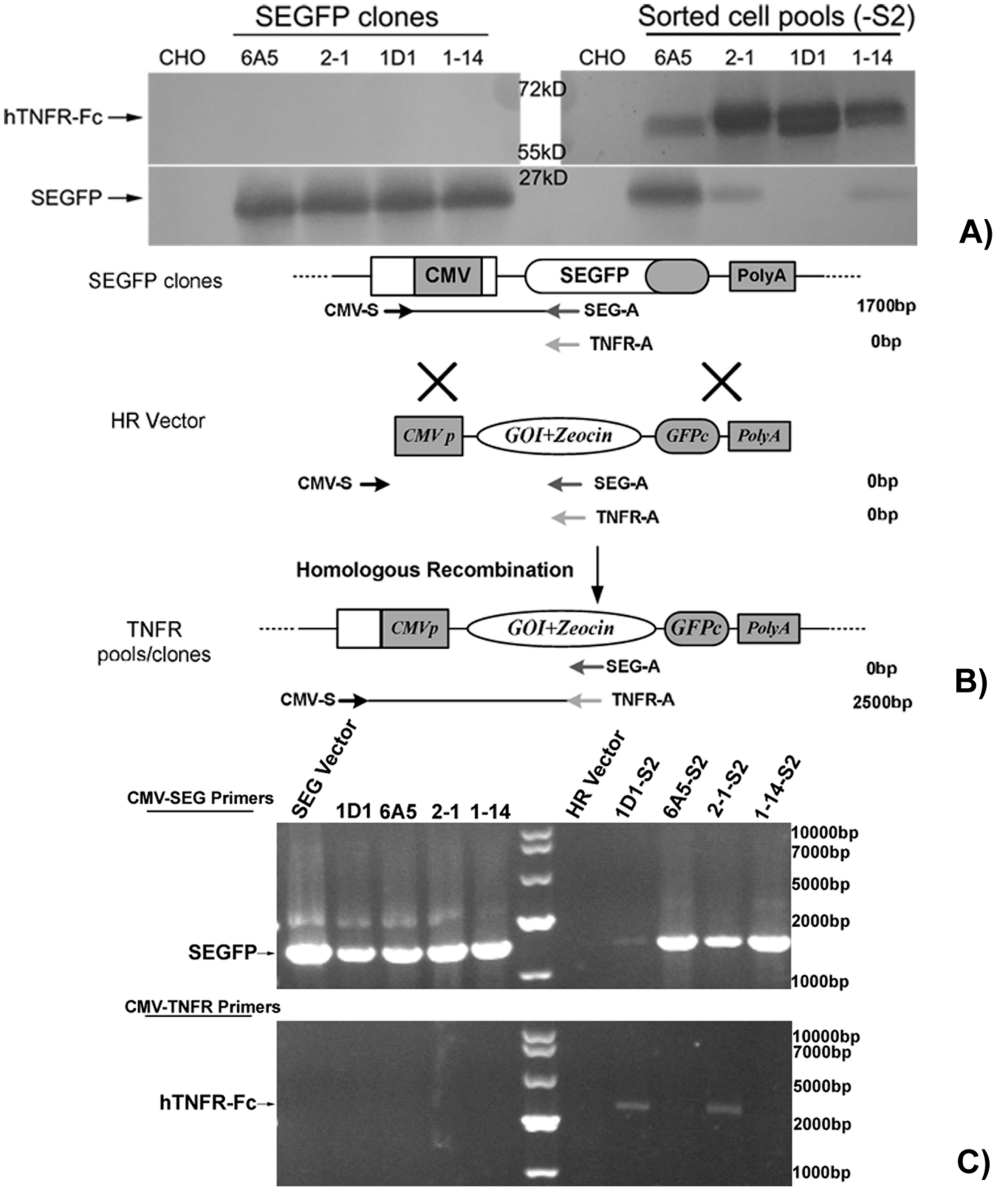
Identification of the GOI expression cassette integrated into pre-selected sites. (A) Immunoblot analysis of SEGFP and TNFR-Fc. After replacement by TNFR-Fc, SEGFP expression decreased in the sorted cell pools compared with that in each parental cell clone, but all sorted cell pools produced TNFR-Fc. (B) Outline of the genomic DNA analysis. Three primers were designed to analyze replacement of SEGFP. Integration of the GOI into a pre-selected site of SEGFP integration was demonstrated by band-shift. (C) Examination of the remaining (silenced) parental cassettes in SEGFP cell clones before (as a positive control) and after exchange (-S2 cell pools). SEGFP and HR vectors were used as controls for SEGFP cell clones and sorted cell pools, respectively.

In accordance with the design, SEGFP-replaced cells would be SEGFP-negative cells, but this assumption was incorrect. Therefore, even though 1D1-S2 and 1-14-S2 cell pools were almost completely composed of GFP-negative cells, as detected by flow cytometry, we also determined whether these cells were replaced cells by another detection method. Consequently, medium supernatants of all four cell pools were collected and analyzed by western blotting. As shown in Fig. 5A, only SEGFP was expressed in the four SEGFP cell clones, and the expression level was consistent with their FI. Moreover, only one band for SEGFP was detected in the medium supernatants of the four sorted cell pools, suggesting that SEGFP was not replaced by the different expression cassettes by HR. Consistent with the flow cytometry results, the SEGFP expression level decreased in all sorted cell pools. Notably, in the 1D1-S2 cell pool, no observed SEGFP band suggested that the SEGFP expression cassette was replaced completely. On the other hand, in all sorted cell pools, TNFR-Fc was expressed at different levels.

To confirm whether HR was complete, we isolated the genomic DNA of SEGFP cell clones and the corresponding sorted cell pools. The reporter gene and GOI were then detected by PCR. As shown in Fig. 5B, we designed three primers for gene amplification. The same upstream primer was used to amplify SEGFP and GOI in the respective DNA samples. It was designed to match the upstream CMV promoter in the SEGFP vector other than the HR vector. Thus, in SEGFP cell clones, the DNA fragment (1700 bp) from the CMV promoter to the C-terminal of SEGFP was amplified using a CMV-S and SEGFP-A primer pairs; without TNFR expression cassette, no band was detected in these genomic DNA using CMV and TNFR-A primers. However, after HR and cell sorting, the DNA band was shifted in site-integrated cells, in which the DNA fragment of CMV-TNFR (2500 bp) was amplified using CMV and TNFR-A primers. Because of mismatching with the sense primer of the CMV promoter, no band was amplified from the HR vector.

In accordance with the flow cytometry and western blot results, the corresponding fragments were amplified from SEGFP cell clones using the CMV-S and SEGFP-A primer pair (Fig. 5C). Different patterns were revealed in different sorted cell pools. The shifted band (2500 bp) was detected only in 1D1 and 2-1 cell pools using CMV and TNFR-A primers, suggesting that the GOI cassette was inserted into the pre-set site of genomic DNA of 1D1 and 2-1 cell clones. However, no specific bands were observed in 6A5-S2 and 1-14-S2 cell pools, indicating that the GOI cassette did not replace the SEGFP site exactly. On the other hand, SEGFP was amplified in all sorted cell pools using CMV-S and SEGFP-A. Some SEGFP-positive cells in 6A5-S2 and 2-1-S2 cell pools may be the reason for this result. Detection of SEGFP in the 1-14-S2 cell pool indicated different HR patterns. The unexpected results of the 1D1-S2 cell pool may have been caused by a few cells containing the SEGFP gene that was not expressed.

### Increased GOI Expression by Integration in Pre-selected Sites

#### Higher GOI expression in the SEGFP-negative cell pool than that in the SEGFP-positive cell pool

**Figure 6:**
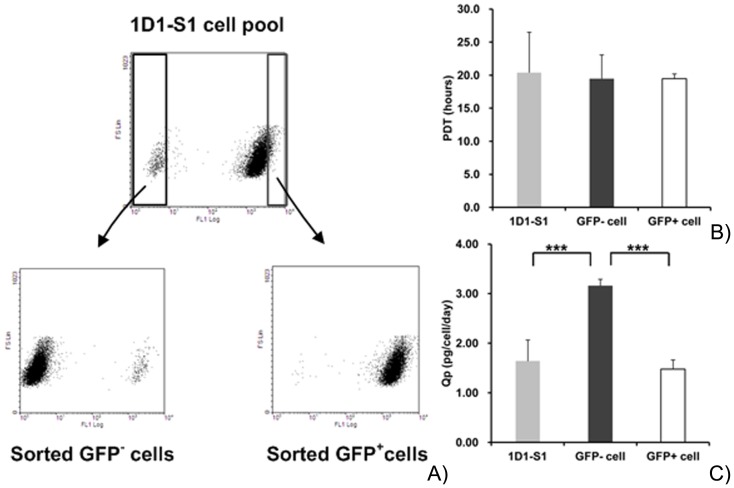
Comparison of site-specific and random integration. (A) Enrichment process of site-integration cells (GFP^−^ cells) and random-integration cells (GFP^+^ cells). The dot plot shows the two subpopulations sorted from the 1D1-S1 cell pool. (B, C) PDT and Qp of TNFR-Fc were compared between the subpopulations. The 1D1-S1 cell pool was used as a control.

To compare the expression level of the GOI with site-specific or random integration, SEGFP-negative cells (GFP^−^ cells) and SEGFP-positive cells (GFP^+^ cells) were separated by FACS (Fig. 6A). As shown above, the GOI in the 1D1-S2 cell pool was integrated into the genomic DNA at a pre-selected site. Thus, the sorted GFP^−^ and GFP^+^ cells represented site-integrated and randomly integrated cells, respectively. Using the 1D1-S1 cell pool as a control, the cell growth and GOI titer of sorted cell pools were compared. The average PDT of the cell pools was approximately 20 h (Fig. 6B), indicating that cell growth had not been inhibited by SEGFP and/or GOI expression. However, the averaged Qp of the GOI in SEGFP-negative cells was significantly higher than that in SEGFP^+^ cells and the 1D1-S1 cell pool (Fig. 6C). These results suggested that the GOI integrated into the high-expression site will produce more target protein than that of the randomly integrated GOI.

#### GOI expression increases in cell pools and clones generated by HR

**Figure 7:**
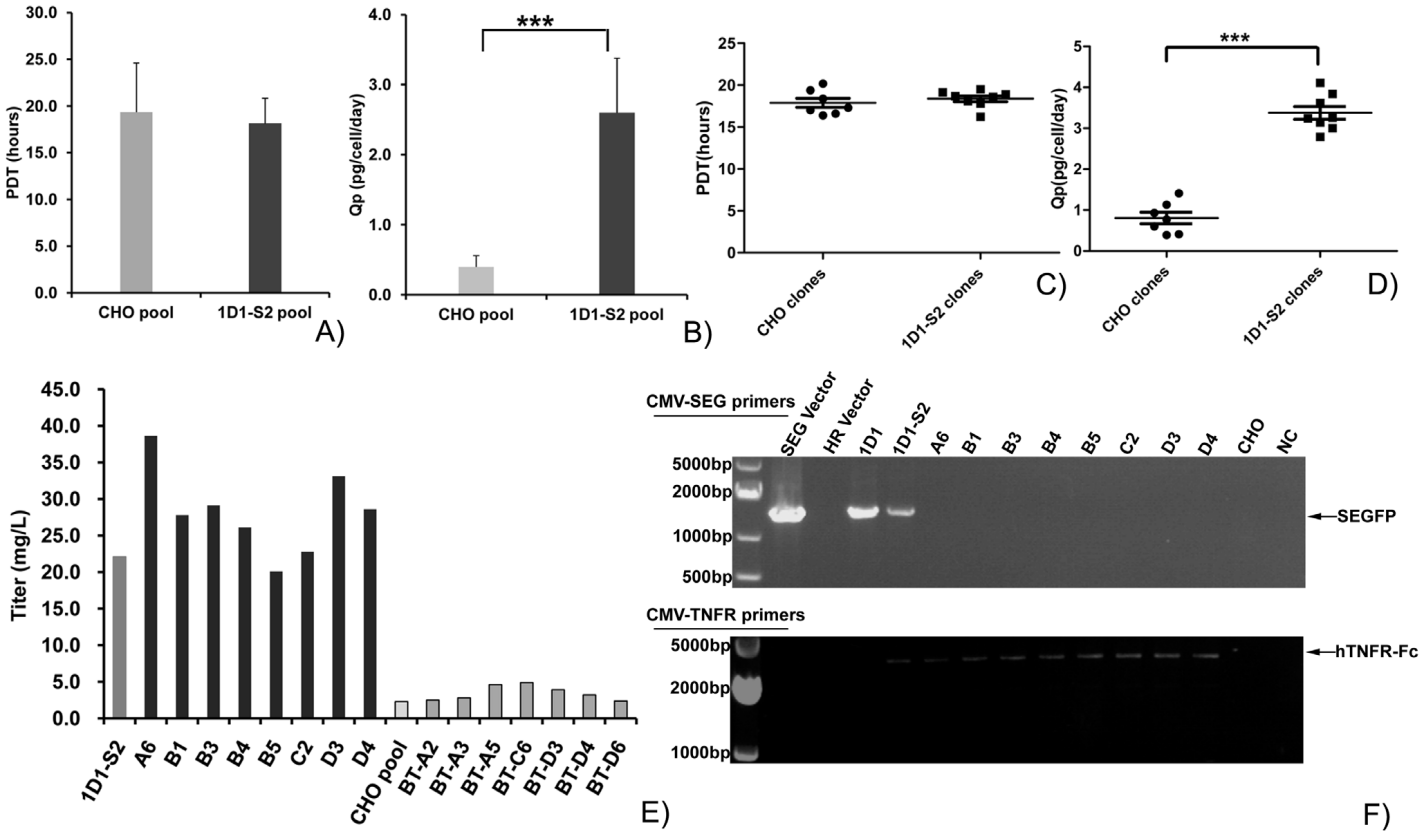
HR strategy to generate cell lines with higher expression of the GOI. (A–E) Compared with the cell pools and clones derived by traditional selection, the growth of the cell pool and single cell clones selected by the HR strategy remained stable (A, C). In contrast, the Qp and total titer of TNFR-Fc was significantly increased in cell pools and clones generated by the HR method (B, D, E). Black columns are cell clones obtained by the HR strategy; light gray columns are cell clones selected by the traditional method (E). (F) Verification of authentic exchange events for the two cell clones derived by the HR strategy. PCR was performed on 1D1 and 1D1-S2 cell pools and all 8 single-clones with primer pairs as indicated in Fig. 5B. Negative controls were CHO cells and H_2_O. Positive controls were SEGFP and HR vectors. The band-shift in all cell clones indicated the SEGFP could be replaced by TNFR-Fc by site-integration exactly.

To verify that the cells with the GOI integrated into the pre-selected site (1D1-S2 cell pool) can express a higher amount of target protein, we transfected HR plasmid C into CHO cells and generateda cell pool by traditional drug selection (CHO cell pool). Cell growth and Qp of both cell pools were compared during subculture in shaking flasks. The PDTs of the cell pools were not significantly different, although the PDT of the 1D1-S2 cell pool (18.1 h) was slightly lower than that of the CHO cell pool (19.3 h) (Fig. 7A).On the other hand, as shown in Fig. 7B, the Qp value of the 1D1-S2 cell pool was approximately 2.6 pg/cell/day, which was more than 5-fold higher than that of the CHO cell pool (0.4 pg/cell/day).

Cell pools generated by the traditional method were composed of a large number of heterogeneous cells with different expression levels of the GOI. Therefore, the advantage of HR could not be demonstrated by comparing cell pools. Forty-six and 42 cell clones were then selected by subcloning CHO and 1D1-S2 cell pools, respectively. According to high GOI expression, seven CHO cells clones and eight 1D1-S2 cell clones were expanded. As shown in Fig. 7C, the GOI in 1D1-S2 cell clones was expressed significantly higher than that in cell clones derived from the CHO cell pool. The Qp values of 1D1-S2 cell clones was 2.8–4.1 pg/cell/day, which was slightly higher than that of the 1D1-S2 cell pool. The PDTs were equivalent among cell clones from different cell pools (Fig. 7D). TNFR-Fc titers of cell clones selected from the 1D1-S2 cell pool were much higher than those of CHO cell clones. Notably, the TNFR-Fc titer of the best 1D1-S2 cell clone was 38.6 mg/L, which was approximately 8-fold higher than that of the best CHO cell clone (Fig. 7E). On the other hand, among cell clones, only the GOI gene was amplified by CMV and TNFR-A primers, and no SEGFP gene fragment was detected by CMV and SEGFP-A primers in All 8 single cell clones (Fig. 7F). The band shift showed an exact gene replacement between the reporter gene and GOI. In conclusion, cell clones from the 1D1-S2 cell pool showed higher GOI expression, and they were composed by cells without the SEGFP gene.

## Discussion

In this study, we present an alternative approach for the generation of high-expression engineered cell lines. Stable cell lines with significantly increased expression of TNFR-Fc were established by combining HR and FACS (Fig. 7E). Furthermore, the cell pool derived from our strategies can express a GOI at a high level, which can be used to produce recombinant protein. This strategy also provides time and cost advantages over traditional processes.

In the biopharmaceutical industry, stability and high-expression, which result from GOI insertion into a genomic hotspot [5], are two critical aspects for engineered cell lines. Our strategy fist established a parental cell line with genomic hotspots indicated by a reporter gene. HR was then used to insert the GOI into the pre-selected site to generate the engineered cell line. Some guiding principles can be summarized from our results of parental cell line generation and the following HR.

In general, the highest expression of the reporter gene in the parental cell line was acquired in batch culture. It has been reported that an increase in specific productivity directly correlates with the cell size and protein content [21], [22]. Our flow cytometry data also showed that cell clones with a higher cell content and larger cell size had a higher QFI (data not shown). However, the titer of a target protein in batch culture is determined by cell density as well as Qp. Porter et al. reported that clones with the highest GOI expression are those with a higher Qp and the highest visual cell density rather than those with the highest Qp [23]. There was only a 15% difference of total FI in our four clones, among which there was an approximate 2-fold difference of QFI. Thus, selection of the parental cells should balance the Qp and cell growth. On the other hand, HR results suggested that the cell clones, such as 1D1 and 1–14, with a higher QFI, high cell density, and shorter PDT may have an advantage for gene replacement.

The replaced cells displayed a consistent cell size and protein content compared with that of their parental cells (Fig. S1). Therefore, the stability of the parental cells was important for the stability of the engineered cell lines, including the stability of cell growth and protein production. These stable cell clones were all single cell clones with significant homogeneity of the cell population, resulting in little difference of antibody or Fc-fusion protein expression in the cell population [13]. In parental cells, the homogeneity of SEGFP expression was easily determined by flow cytometry. 1D1 and 1–14 cell clones had better homogeneity than that of the other two clones (Table 1). On the other hand, the 6A5 cell clone had more heterogeneous cells with significant instability of cell growth and SEGFP expression. Moreover, cell clones of GOI, derived from 1D1-S2 pool, exhibited similar characters, such as Qp, PDT and HR patterns (Fig. 7D–F). Thus, homogeneity is an important character for selection of parental cells to generate stable gene-replaced cells for both cell growth and GOI expression. Following these principles, much more SEGFP cell clones were selected in the next round. The cell clones of GOI with higher-expression and extract HR replacement were generated by using those SEGFP parental cell clones, similar with 1D1 (data not shown).

Copy number of the reporter gene in parental cells is also an important factor. In theory, a single copy of the reporter gene is required for gene replacement with the GOI [19]. More than one copy of the reporter gene will significantly reduce the HR efficiency [24]. However, our results indicated that not only a single copy of the reporter gene can be replaced by HR; it has also been reported that high-expression cell lines can be established by HR of a parental cell line containing two copies of the reporter gene [25]. Therefore, to increase the efficiency of HR and obtain more replaced cells, the parental cells should contain the lowest possible copy number of the reporter gene.

In addition to establishment of parental cell lines, construction of the HR vector is also very important. HR plasmid C, which expressed the GOI as well as the control plasmid (Fig. 3B), was used to insert the GOI into the hotspot. Moreover, this construction can increase the GOI expression level when the plasmid randomly integrates into other genomic sites in addition to the pre-selected site. Our results indicated that only a 200 bp homologous region could guide HR between the GOI and reporter gene. Even without recombinase, the 34 bp minimal Flp recognition target (FRT site) can increase the probability of HR (data not shown). One of the reasons is that electroporation can increase the efficiency of DNA importation into the nucleus, which improves the probability of HR [26], [27].

Hasty et al. reported that only 472 bp of homology is used as efficiently as 1.2 kb in the formation and resolution of cross over junctions [28]. However, to obtain HR cell clones, the size of the homologous region has to be more than 1000 bp. Following these principles, HR plasmids A and B were constructed simultaneously with a longer homologous region. However, all constructions can indicate, respectively,similar DNA structure after HR. GOI expression was very low using transient transfection of plasmids A and B, suggested that the GOI may be expressed at a very low level in genomic DNA. To determine whether GOI expression was inhibited by transcription of the SEGFP N-terminal under the full-length CMV promoter, HR plasmids A and B were also improved by inserting the TK polyA sequence following the SEGFP N-terminal sequence. However, the decrease of GOI expression did not recover using the improved plasmid (data not shown). In our study, the efficiency of HR was very low, even though we increased the efficiency using the homologous region. However, using SEGFP as the reporter gene, HR cells should be SEGFP-negative cells that could be easily enriched by FACS. As expected, almost all SEGFP-negative cells in 1-14-S2 and 1D1-S2 cell pools were selected by the two-round sorting process (Fig. 4B). On the other hand, complete HR could not be ensured by SEGFP-negative expression. Integration of the GOI expression cassette in the pre-selected site should be determined by expression of the GOI and analysis of genomic DNA. Analysis of 1-14-S2 and 1D1-S2 cell pools demonstrated that the HR process could result in different replacement patterns. Furthermore, with differences among 1D1-S2 cell clones, the SEGFP gene was also detected in 1-14-S2 cell clones (data not shown). In summary, SEGFP-negative cells can be enriched by FACS, but GOI expression and DNA analysis are needed for exact determination of replaced cells.

Different aspects of the engineered cell line establishment need to be improved, although high-expression cell lines can be selected with our strategy. First, it has been reported that the S/MARs region can increase reporter gene expression, and increase expression of a GOI inserted by RMCE. However, the limitations of gene expression with a single copy are unknown. In addition, the expression level of the reporter gene in the parental cell line should be as high as possible. However, accumulation of the reporter gene may affect cell growth. Our use of SEGFP would decrease the accumulation of intracellular GFP, and then reduce the inhibiting effect of GFP on cell clone selection and cell growth. However, SEGFP would also be expressed in cells, which may affect selection of high-expression cells. Thus, to indicate the best parental cell line with a high-expression site, we need to improve the expression cassette of the reporter gene or construct a more suitable reporter gene.

## Supporting Information

Figure S1 **Cell size and protein content are consistent between the parental cell line and their corresponding sorted cell pools (-S2).** FS: Semi-quantitative granularity profiles from FACS analysis of the forward scatter of at least 10,000 cells; SS: Semi-quantitative granularity profiles from FACS analysis of the side scatter of at least 10,000 cells. CHO cells were used as the control.
(TIF)
