# Supplementary figures and images for: Combination of FACS and Homologous Recombination for the Generation of Stable and High-Expression Engineered Cell Lines
Source: PLoS One. 2014 Mar 19;9(3):e91712. doi: 10.1371/journal.pone.0091712 (PMC3960159; doi:10.1371/journal.pone.0091712)

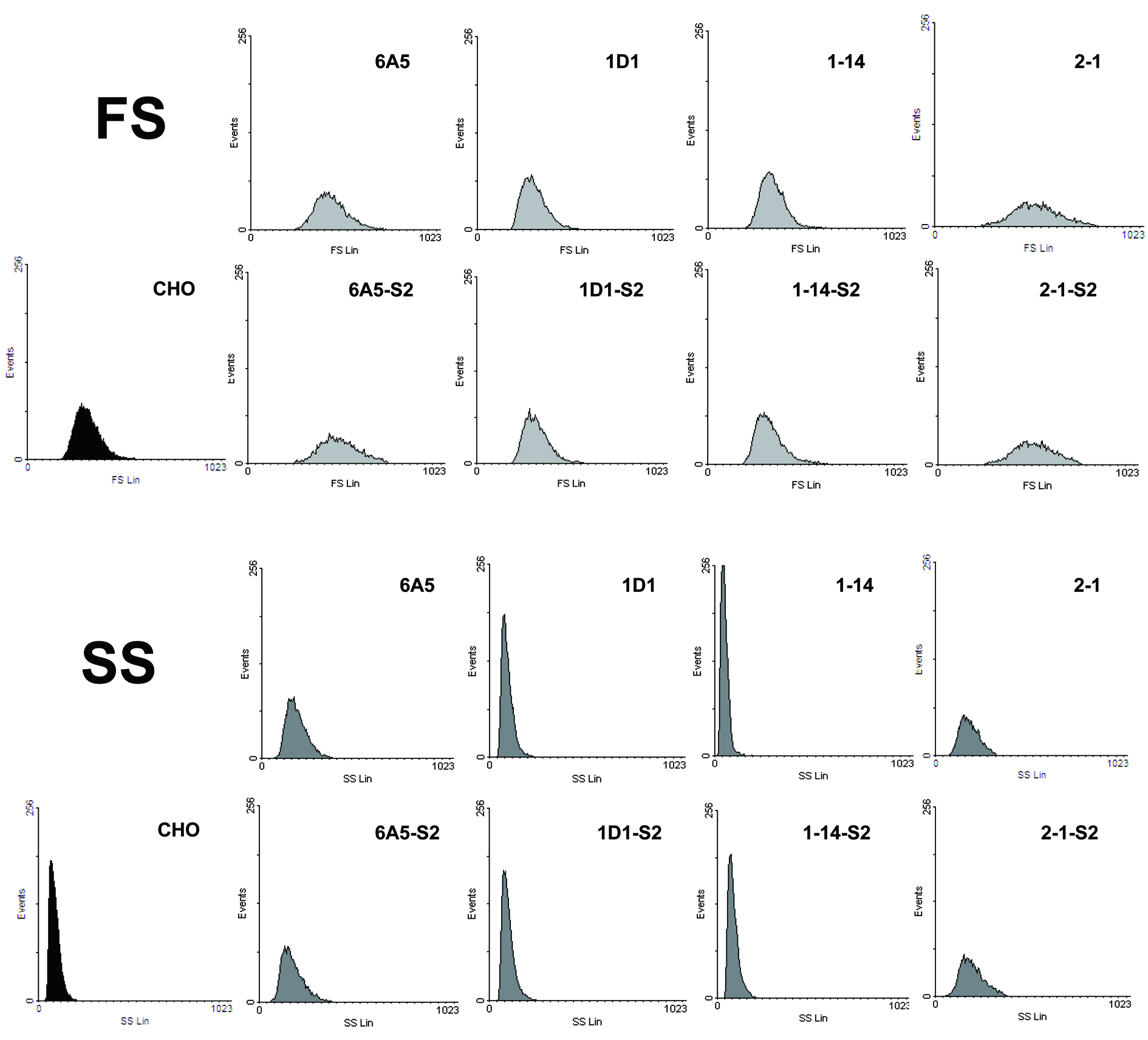

Supplement: Figure S1 — Cell size and protein content are consistent between the parental cell line and their corresponding sorted cell pools (-S2). FS: Semi-quantitative granularity profiles from FACS analysis of the forward scatter of at least 10,000 cells; SS: Semi-quantitative granularity profiles from FACS analysis of the side scatter of at least 10,000 cells. CHO cells were used as the control. (TIF) [file pone.0091712.s001.tif]
